# Impact of Introducing the Line Probe Assay on Time to Treatment Initiation of MDR-TB in Delhi, India

**DOI:** 10.1371/journal.pone.0102989

**Published:** 2014-07-24

**Authors:** Neeta Singla, Srinath Satyanarayana, Kuldeep Singh Sachdeva, Rafael Van den Bergh, Tony Reid, Katherine Tayler-Smith, V. P. Myneedu, Engy Ali, Donald A. Enarson, Digamber Behera, Rohit Sarin

**Affiliations:** 1 National Institute of Tuberculosis & Respiratory Diseases, Delhi, India; 2 International Union against Tuberculosis & Lung Disease, South-East Asia Regional Office, New Delhi, India; 3 Central TB Division, Directorate General of Health Services, Ministry of Health and Family Welfare, Government of India, New Delhi, India; 4 Médecins Sans Frontières – Operational Centre Brussels, Operational Research Unit (LuxOR), Luxembourg; 5 International Union against Tuberculosis & Lung Disease, Paris, France; 6 Postgraduate Institute of Medical Education & Research, Chandigarh, India; Institut de Génétique et Microbiologie, France

## Abstract

**Setting:**

National Institute of Tuberculosis and Respiratory Diseases (erstwhile Lala Ram Sarup Institute) in Delhi, India.

**Objectives:**

To evaluate before and after the introduction of the line Probe Assay (LPA) a) the overall time to MDR-TB diagnosis and treatment initiation; b) the step-by-step time lapse at each stage of patient management; and c) the lost to follow-up rates.

**Methods:**

A retrospective cohort analysis was done using data on MDR-TB patients diagnosed during 2009–2012 under Revised National Tuberculosis Control Programme at the institute.

**Results:**

Following the introduction of the LPA in 2011, the overall median time from identification of patients suspected for MDR-TB to the initiation of treatment was reduced from 157 days (IQR 127–200) to 38 days (IQR 30–79). This reduction was attributed mainly to a lower diagnosis time at the laboratory. Lost to follow-up rates were also significantly reduced after introduction of the LPA (12% versus 39% pre-PLA).

**Conclusion:**

Introduction of the LPA was associated with a major reduction in the delay between identification of patients suspected for MDR-TB and initiation of treatment, attributed mainly to a reduction in diagnostic time in the laboratory.

## Introduction

Among patients with multidrug resistant- tuberculosis (MDR-TB), delays in diagnosis and treatment initiation are frequently observed, resulting in an increased risk of disease complications and high mortality and pre-treatment lost to follow-up rates. In addition, such delays lead to an extended period of TB infectivity within the community, resulting in higher transmission rates, higher mortality and morbidity [1].

As in-vitro confirmation of resistance to Isoniazid and Rifampicin (the two most important first line anti-TB drugs) is essential for the diagnosis of MDR-TB, a major reason for the delay in starting MDR-TB treatment is the prolonged time taken by the laboratory to make the diagnosis of drug resistance [2]–[4]. Most national tuberculosis programme laboratories in high TB burden countries use either solid or liquid culture media for drug sensitivity testing (DST) which respectively take on an average 84 and 42 days to make a diagnosis [5], [6].

India is one of the highest TB burden countries in the world with about 64,000 estimated cases of MDR-TB annually [7]. India began implementation of programmatic management of drug resistant tuberculosis in 2007 and has gradually expanded the services nationwide. However progress was slow due to limited laboratory capacity and long turn-around times using established diagnostic methods described earlier [7]–[9]. In 2011, the Line Probe Assay (LPA) was introduced as a new diagnostic technique in the Indian National TB Programme. It is a method based on nucleic acid amplification, permitting rapid detection of mutations in genes coding for resistance to Rifampicin and Isoniazid (Hain test). The LPA test only requires an average time of two days to diagnose MDR-TB, which is vastly shorter than the previous diagnostic methods [9]–[12]. Currently there are 41 laboratories nationwide implementing the LPA tests and the country plans to establish more laboratories with LPA testing facilities. Shortening the time to diagnosis of MDR-TB has the potential to improve access to appropriate treatment and reduce losses to follow-up. To date however, there is no published literature documenting the associated programmatic impact of introducing LPA for MDR-TB diagnosis in India.

In Delhi, India, we therefore compared the following parameters before and after the introduction of the LPA: a) the overall time interval between suspicion of patients for MDR-TB and their treatment initiation and b) the time intervals for four different steps of patient management between suspicion of patients for MDR-TB and treatment initiation. Additionally, we compared pre-treatment losses to follow-up rates before and after introduction of the LPA test.

## Methods

### Design

This was a retrospective cohort study using routinely collected programme data.

### Setting

At the time of the study, Delhi state, with a population of 16.8 million and an estimated TB burden of 55,000 patients had two certified laboratories for the diagnosis of MDR-TB, both of which introduced the LPA in October 2011. Before this date, the laboratories were using solid culture and DST for the patients under the National programme. There are four Drug Resistant TB Centres (DR-TB centres) for initiating MDR-TB treatment and managing diagnosed MDR-TB patients. One of these diagnostic laboratories and the associated DR-TB centre is located at the National Institute of TB & Respiratory Diseases (NITRD, erstwhile Lala Ram Sarup Institute). The laboratory of the institute is also one of the National Reference Laboratory for the National TB Programme in India.

### Diagnosis and treatment of MDR-TB

From 2009 till September 2011, the criteria for suspecting MDR-TB under the national programme were: treatment failures among new TB cases, smear positive cases that remained smear positive after the fourth month of treatment with Retreatment regimen, and pulmonary TB cases who were contacts of known MDR-TB cases (Criteria A-Pre LPA cohort). During this period, solid culture (LJ Media) and drug sensitivity testing (1% proportion method) was done [13]. From October’11, with introduction of rapid diagnostic method (LPA) the criteria for presuming MDR-TB were expanded to also include all smear positive and negative previously treated pulmonary TB cases at diagnosis, any smear positive follow-up results in new or previously treated cases, and all HIV/TB co-infected cases at diagnosis (Criteria B & C-Post LPA cohort). During this period, the LPA (Hain test) was done; a molecular test which identifies mutations conferring resistance to Rifampicin and Isoniazid. This test was done only on smear positive samples [14]. All sputum negative samples were put on liquid culture (MGIT) and LPA was done from these cultures.

TB patients who fulfilled the criteria for suspecting MDR-TB were identified in the peripheral DOT centres by medical officers and lab technicians, and referred to the lab of the National Institute of TB and Respiratory Diseases for diagnosis and further management. All patients diagnosed as resistant to Rifampicin and Isoniazid (MDR-TB) or resistant to Rifampicin and sensitive to Isoniazid were referred to a Drug Resistant TB Centre for initiation of MDR-TB treatment. At this Drug Resistant TB centre, patients were hospitalised and a pre-treatment evaluation was performed: treatment was initiated after approval by the Drug Resistant TB Centre committee. Treatment of MDR-TB under the National TB programme is the standard drug regimen comprising of Intensive phase of 6 to 9 months of Kanamycin, Livofloxacin, Ethionamide, Cycloserine, Pyrazinamide, and Ethambutol followed by continuation phase of 18 months of Livofloxacin, Ehionamide, Cycloserin, and Ethambutol. PAS is used as a substitute drug.

### Inclusion criteria and study population

The study inluded all patients enrolled between January 2009 and December 2012 at the NITRD laboratory who met the following criteria: a) diagnosed as MDR-TB either by solid/liquid culture & DST or LPA at the Institute’s laboratory, b) were either pretreatment smear positive retreatment patients or had positive follow-up sputum smears while on new or retreatment regimens, c) started treatment from the NITRD DR-TB centre, and d) had a complete record of dates at the time of data collection. Patients enrolled between January 2009 and September 2011 comprised the pre-LPA group. The post-LPA phase was from October 2011 and December 2012. For the post-LPA period, to assess various time periods between suspecting MDR-TB to treatment initiation, we collected data for 2 consecutive quarters from October 2011 to March, 2012 with cut-off date of 30th March 2012. During the pre-LPA phase, a total of 121 patients were initiated on treatment, and of these 51 were included in the study based on the inclusion criteria. Patients enrolled between October 2011 and December 2012 comprised the post-LPA group. During this phase, 433 patients were enrolled on MDR-TB treatment, and of these 83 were included in the study based on inclusion criteria and cut-off date. The time taken from suspecting MDR-TB in patients to initiation of MDR-TB treatment at the Drug Resistant TB centre was evaluated in each group.

To compare the pre-treatment lost to follow-up rates between the pre- and post-LPA phase, data on all patients suspected of MDR-TB identified between January 2009 and December 2012 were collected.

### Data sources, variables and definitions

Data on the socio-demographic characteristics of the study population were sourced from the referral for culture DST Register, laboratory register and TB register for MDR-TB patients. The data included the following data variables: age, sex, type of TB, and dates pertaining to diagnosis and treatment initiation.

Data to examine the time intervals between MDR-TB suspicion and treatment were sourced from the Revised National Tuberculosis Control Programme records (TB treatment cards, Referral for Culture & DST forms and Drug Resistant TB register). The main study variables included the dates of suspecting MDR-TB in the patient, laboratory referral, diagnosis, and treatment initiation; The total time from date of suspecting MDR-TB to initiation of treatment was sub-divided as: a) time from suspecting MDR-TB to laboratory referral; b) time from the specimens reaching the laboratory to MDR-TB confirmation; c) time from MDR-TB confirmation to presentation at the DR-TB centre; and d) time from presentation at the DR-TB centre to MDR-TB treatment initiation.

Data to assess lost to follow-up were sourced from Referral for culture and DST register, Laboratory reports and Drug resistant TB (DR-TB)registers; Patients diagnosed as MDR-TB but not enrolled for treatment in DR-TB register were considered lost to follow-up. All data were collected retrospectively.

### Data validation, entry and analysis

Data validation was done by comparing the data from MDR-TB register, individual treatment cards and treatment files, and cross-checking all the records. Data were double-entered intoEpiData v.3.1 (EpiData Association, Odense, Denmark), and cross-verified for consistency.

Data were analysed using EpiData Analysis software v.2.2.1.171 (EpiData Association Odense, Denmark): simple summary statistics were calculated, and differences between groups were compared using Chi-square test and Kruskal Wallis test as appropriate. A *p*-value <0.05 was taken as statistically significant. Data concerning the pre-treatment lost to follow-up rates were extracted from the quarterly programme reports of Drug Resistant TB Centre of NITRD.

### Ethics

This study was reviewed and approved by the Institutional Ethics Committee of the National Institute of TB and Respiratory Diseases, New Delhi, India. In addition, this study met the Médecins Sans Frontières’ Ethics Review Board (Geneva, Switzerland)-approved criteria for analysis of routinely-collected programme data and was also approved by the Ethics Advisory Group of the International Union against Tuberculosis and Lung Disease, Paris, France. As this study involved review of routinely collected patient data from hospital records under confidentiality, the ethics committees waived us from obtaining written consent from patients.

## Results

A total of 51 and 83 patients fulfilled the inclusion criteria for assessment of the time taken from suspecting MDR-TB to initiation of MDR-TB treatment for the pre- and post-LPA study periods respectively. There were no differences in the demographic characteristics of these two groups **(** **Table 1** **).**

**Table 1:** Demographic characteristics of MDR-TB patients according to the method of MDR-TB diagnosis used between 2009–2012 - New Delhi, India.

*Variable*	Pre-LPA^a^	Post-LPA^b^	
	n (%)	n (%)	*P*-value
**Total**	51	83	
**Sex**			
Male	29(57)	54(65)	0.34
Female	22(43)	29(35)	-
**Age (years)**			
<35	32(63)	59(71)	0.31
≥35	19(37)	24(29)	-
Median, years (IQR)	30(22–40)	27(21–37)	0.31
**TB category**			
New case	10(20)	16(19)	0.96
Previously treated	41(80)	67(81)	-

a Solid culture & Drug Sensitivity Testing (DST),

b LPA: Line Probe Assay, MDR-TB: Multidrug resistant Tuberculosis, IQR: Inter-quartile Range.

The overall median time from suspecting MDR-TB to the initiation of treatment was reduced from 157 (IQR 127–200) days to 38 days (IQR 30–79), shown in **Table 2**. The major reason for this reduction was the time taken by the laboratory to diagnose MDR-TB and provide the results, which decreased from 107 days (IQR 79–131) to 5days (IQR 3–6) (Step 2). The intervals between identification of presumed MDR-TB cases and referral to the laboratory for specimens decreased from 12 days (IQR 7–29) to 9 days (IQR 4–31) (Step 1) and between laboratory notification and patients reporting to the Drug Resistant TB Centre from 12 days (IQR 4–26) to 7 days (IQR 5–13) (step 3), but the delays were not significantly different pre-and post-LPA. The time taken by the Drug Resistant TB centre to initiate treatment increased from 8 days (IQR 7–13) to 12days (IQR 9–17) (step 4).

**Table 2:** Comparison of time from identification of patients suspected for MDR-TB to initiation of MDR-TB treatment between pre- and post-LPA period - New Delhi, India.

Steps between identification of patients suspected for MDR-TB and treatment initiation	Median time, days (IQR)		*P*-value*
	Pre-LPA^a^	Post-LPA^b^	
Step 1: Time from identification of patients suspected for MDR-TB to submission of samplesin the laboratory	12(7–29)	9(4–31)	0.07
Step 2: Time taken in the laboratory to test and provide the MDR-TB report	107(79–131)	5(3–6)	<0.0001
Step 3: Time taken from laboratory report to patient reporting to Drug Resistant TB centre	12(4–26)	7(5–13)	0.3
Step 4: Time taken by Drug Resistant TB centre to initiate treatment	8(7–13)	12(9–17)	0.006
TOTAL (Step 1–Step 4)	157(127–200)	38(30–79)	<0.001

*P-value generated using the Wilcoxon rank sum test; IQR = Inter-quartile range; MDR-TB = Multidrug resistant Tuberculosis;

a Solid/liquid culture & Drug Sensitivity Testing (DST).

b LPA: Line Probe Assay.

**Figure 1:**
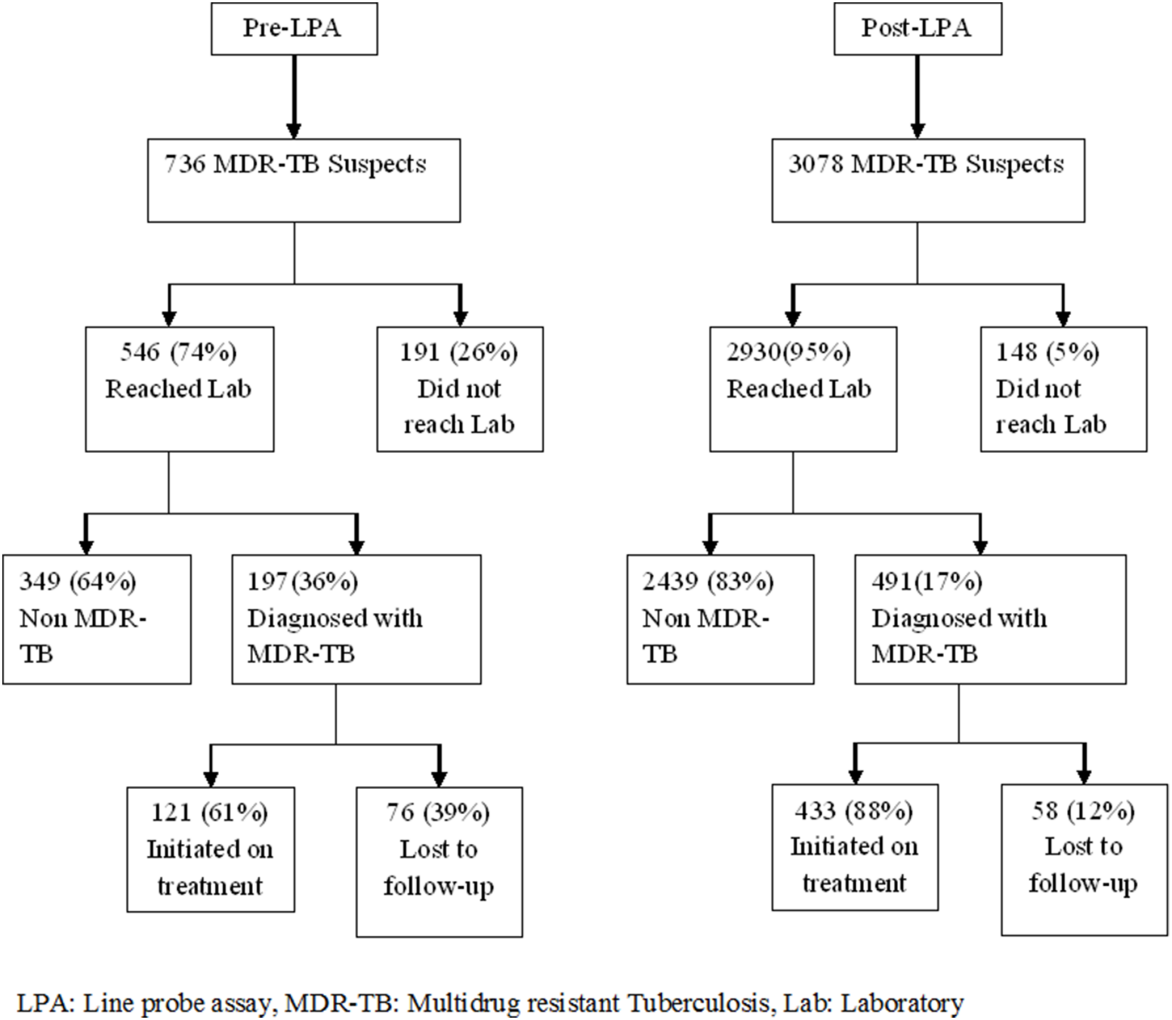
Pre-treatment loss to follow-up rate among MDR-TB suspects before and after diagnosis is in the pre-LPA and post-LPA periods at New Delhi, India.

The cohort used for pre-treatment lost to follow-up analysis included a total of 736 and 3078 patients suspected for MDR-TB during the pre-LPA and post-LPA periods respectively (**Figure 1**). Post-LPA, the losses were significantly reduced between identification of patient suspected for MDR-TB and specimens reaching the laboratory, and between diagnosis and treatment initiation.

Finally, the overall pre-treatment lost to follow-up from suspicion of patients for MDR-TB to start of treatment was significantly lower during the post-LPA period compared to the pre-LPA period.

## Discussion

We believe that this is first study from India to assess the impact of introduction of LPA on the length of time between identification of presumed MDR-TB, diagnosis of MDR-TB and initiation of treatment under routine programmatic conditions. The introduction of LPA significantly reduced the time taken by the laboratory to diagnose MDR-TB. As a result, the total time taken to diagnose and initiate MDR-TB patients on treatment was reduced to an average of one and a half months from an average of five months previously.

The study has several strengths. First, it was carried out in a tertiary referral institute which is a National Reference laboratory and it had all the standard quality control measures in place. Thus, the reported results of the sputum culture and DST were highly reliable. Second, the study was conducted in a routine programme setting and therefore is likely to reflect the reality on the ground. Finally, data pertaining to the study were validated by us by cross checking multiple registers and we therefore believe that they are robust.

The results of this study are comparable to those reported from South Africa [3] in which 75% of patients with MDR-TB had a delay in diagnosis and treatment of up to 22 weeks leading to prolonged infectivity and morbidity. From a public health perspective, reducing the period between diagnosis and treatment initiation by the introduction of the LPA has direct benefits for both the patient and the community. Patients benefit from early diagnosis and earlier initiation of appropriate treatment which should result in reduced morbidity and mortality. The community benefits from reduced transmission of MDR-TB as a result of reduced duration of infectivity of index patients.

Although the overall delay between suspicion of MDR-TB and initiation of treatment was reduced, the fact that it still takes on average a month and a half for the diagnosis and initiation of treatment for MDR-TB suggests that there is a need to further review and improve the process. First, the identification of persons with suspicion for MDR-TB needs to improve, by maintaining a high level of vigilance and rapid appropriate investigation of patients who are not responding to first line anti-TB treatment. Second, time delays at the laboratory could be further reduced by the introduction of an automated cartridge based nucleic acid amplification test (CB-NAAT, such as Xpert MTB/RIF) that has the potential to diagnose MDR-TB among high risk individuals in a matter of two hours at the laboratory, thereby providing results on the same day. CB-NAAT also requires less laboratory sophistication and human resource expertise than LPA in conducting tests for MDR-TB. Third, paradoxically the time to initiate MDR-TB treatment by the DR-TB centre after diagnosis was found to be longer with the LPA system compared with using the established solid and liquid culture media. One of the possible reasons for this could be that the capacity of the NITRD Drug Resistant TB centre in terms of the number of patients that can be handled remained the same during the entire period. This may have resulted in delays in referring patients for treatment (due to queuing up of patients for hospital admission). This issue needs to be confirmed by a further in-depth study (which was beyond the scope of the present study) and if found to be true, it must be addressed by increasing the number of staff and the capacity of centres to initiate more MDR-TB patients on treatment [15].

Several limitations of the study were also identified. First, although LPA has clearly reduced the time to diagnosis, the reduction in total time taken between diagnosis and treatment could have been influenced by other factors, such as better recording and reporting and the recent introduction of supervisors for MDR-TB at the district level. Second, the data compiled for the lost to follow-up rates are from the routine data produced by the programme. During the LPA period, there may have been patients who were diagnosed by other certified laboratories in Delhi or by solid/liquid culture used elsewhere. However, we think that the number of such cases is likely to be small and is unlikely to impact on the current study results. Third, LPA as a technology for the diagnosis of MDR-TB poses some major operational challenges for its implementation and scale-up in resource-limited settings. The assay can be done only on sputum specimens that are smear positive, it is expensive, it requires sophisticated laboratory equipment, quality control measures must be in place, human resources must be trained and there must be a backup of solid/liquid culture to manage sputum specimens that are smear negative.

### Conclusions and recommendations

The study shows that the introduction of LPA testing has a major impact on the management of MDR-TB in Delhi, India. It reduced the overall time from suspicion of patients for MDR-TB to initiation of treatment by effectively reducing the laboratory diagnostic time. At the same time the Drug Resistant TB Centres may need to increase their capacity to deal with the increased load of MDR-TB patients resulting from introduction of LPA. These findings may help to improve the management and treatment of MDR-TB in India.
